# ‘Social Citizenship’ at the Street Level? EU Member State Administrations Setting a Firewall*

**DOI:** 10.1111/jcms.13028

**Published:** 2020-02-18

**Authors:** Anita Heindlmaier

**Affiliations:** ^1^ University of Salzburg Salzburg

**Keywords:** EU citizenship, court of justice, welfare rights, compliance, member state administrations

## Abstract

European integration, and especially the European Court of Justice, has challenged the national character of social rights; the latter have become increasingly transnational. This contribution examines the impact of the Court at the street level. It analyses how Member State administrations handle the social rights of mobile EU citizens in practice in case they are granted discretion. Therefore, a framework of shades of compliance is developed that captures Member State responses to EU law beyond the dichotomy of compliance and non‐compliance. I argue that Member State administrations tend to make the access to social benefits difficult. Still, there may be differences in the shade of compliance on the ground. Surprisingly, these differences cannot be explained by the party‐political environment but depend to a high degree on exposedness. The claim is empirically supported by a comparative study of Austrian welfare (and migration) administrations' practices.

## Introduction

European integration, and especially the European Court of Justice (ECJ), has challenged the national character of social rights; the latter have become increasingly transnational, also for economically inactive EU citizens (Barnard, 2005; Martinsen and Falkner, 2011). While several scholars held that the ECJ thus created a ‘social Europe’ (Caporaso and Tarrow, 2009), others argued that it weakened national welfare states (Höpner and Schäfer, 2012). Although these scholars of ‘integration through law’ differ with regard to their opinion on the effect of ECJ case law, they all share the opinion that it has significant impact on Member States.

Yet, in order to learn about the Court's impact, one needs to consider the implementation of its case law within Member States (Conant, 2002), and also beyond transposition (Treib, 2014, p. 29): one also needs to study its application. Scholars of Europeanization through case law have recently started to examine the impact of the ECJ in practice (for instance: Conant, 2002; Dörrenbacher, 2017), and researchers have already shown that both sides of integration through law scholars overrated the impact of the ECJ (O'Brien, 2017a, 2017b; Shaw and Miller, 2013; Martinsen *et al.,* 2019).

Still, there are gaps that this contribution seeks to overcome. To begin with, there is need for further analytical specification of the concept of compliance. Scholars in the field had traditionally differentiated between compliance on the one hand and non‐compliance (Börzel, 2001) or contained compliance (Conant, 2002, 32 f.), namely compliance in the specific case but no broader policy change, on the other hand. This dichotomy is not able to grasp all the possible reactions of Member States to ECJ case law, as (not) meeting the law is not always black and white (Blauberger, 2012, p. 111). Moreover, Europeanization through case law has not provided an argument for why there is limited social citizenship and thus a limited impact of the ECJ at the street level. The article seeks to overcome these gaps by addressing how Member State authorities handle ECJ case law on social citizenship in practice. And how can variation on the ground be explained? The article focuses on how local administrations, in case they are *granted* discretion and confronted with vague (legal) provisions, *use* this discretion (Hupe and Buffat, 2014, p. 551).

The contributions of this article are thus threefold; first, I develop shades of compliance which capture Member State behaviour besides the current dichotomy of compliance and non‐compliance. Second, I argue that, if the impact is not already clearly limited at a political and guiding level via signalling (Martinsen *et al.,* 2019), local Member State administrations have tended to limit the influence of the ECJ entailing a shade of compliance in terms of ‘less Europe’. This can be explained by the fact that, in the end, administrations share the same rational incentives, to keep both administrative and financial costs low. Still, there may be variation in the degree of ‘(non‐)compliance’ between and *within* Member States. Third, these differences on the ground cannot be explained by the party‐political environment (contrary to the findings of Keiser, 1999; Treib, 2004), but rather depend on exposedness (Martinsen, 2005), namely a high amount of (similar) requests. Empirically, I illustrate my argument with comparative case studies on Austrian local practices concerning the access of economically inactive EU citizens (who reside between three months and five years in Austria) to minimum benefits since 2006. 1In 2006, Directive 2004/38 had to be implemented into national legislation. The case studies mostly build upon interviews with local welfare and migration authorities.

In the subsequent section, I present the relevant legal framework, including the criteria established by ECJ case law under which economically inactive EU citizens are entitled to social benefits. Afterwards, I explicate my analytical framework of shades of compliance and the argument accounting for the limited impact of the ECJ in general and variation on the ground. The case studies on Austrian local practices illustrate my argument. The final section concludes.

## I. The Legal Framework: ECJ Case Law on EU Social Citizenship

EU citizens' rights of free movement and equal treatment derive from the Treaties and their interpretation by the Court, and are further enunciated in secondary legislation, most importantly Directive 2004/38. A key characteristic of Union law on social citizenship is that it is complex and characterized by underspecified core concepts (Davies, 2016, p. 4), as will be explicated hereafter.

Based upon the principle of non‐discrimination on grounds of nationality (Art. 18 Treaty on the Functioning of the European Union, TFEU), European integration, and in particular the European Court of Justice had extended the right to move freely within the EU. While it was originally solely granted to workers, persons finally enjoyed it simply as a ‘citizen of the Union’ (Art. 21 TFEU) with the Maastricht Treaty. The Court linked this concept of Union citizenship again to the principle of non‐discrimination, leading also to wide, but complex social rights for non‐active EU citizens.

A key point of ECJ rulings was that Member States had to show a ‘certain degree of financial solidarity’ between nationals and mobile EU citizens (*Grzelczyk*, C‐184/99). However, EU citizens could not become a burden on the finances of the Member State of residence: Directive 2004/38 codifies earlier case law and specifies the limitations and conditions of free movement rights. While it generally grants economically inactive EU citizens the right to equal treatment as soon as they reside more than three months in the host Member State (Recital 20, Art. 24 Directive 2004/38), it clarifies that those persons are only allowed to reside longer than this period if they have a health insurance and ‘sufficient resources […] not to become a burden on the social assistance system of the host Member State’ (Art. 7 Directive 2004/38). Sufficient resources, for their part, are also only vaguely defined (Minderhoud, 2016): Member States are not allowed to fix a clear amount but have to consider the individual situation of the respective person. In any case, the amount shall not be higher than the threshold for social assistance (Art. 8(4) Directive 2004/38). If economically inactive EU citizens draw social assistance benefits, it is possible that they will no longer meet the criteria for lawful residence, and Member States can terminate their residence, but only if the person has become an ‘unreasonable burden’ on their social assistance system. An expulsion measure shall, thus, not be the *automatic* consequence of an EU citizen's recourse to social assistance (Art. 14 Directive 2004/38).

In its rulings, the ECJ emphasized that Member States had to assess each benefit request of economically inactive EU citizens individually and to apply the principle of proportionality (Barnard, 2005, p. 215). From *Martínez Sala* (C‐85/96) to *Brey* (C‐140/12), different factors were finally decisive for equal treatment, for instance, a certain degree of integration or the fact that people were not becoming an unreasonable burden on the social assistance system of the respective country (Wollenschläger, 2011; Thym, 2015). EU law hence provided that Member States could consider factors warranting a genuine link as part of the assessment of different individual situations (O'Brien, 2017a, 2017b).

Crucial element of ECJ case law is that it is ‘very detailed and indeterminate at the same time’ (Blauberger and Schmidt, 2017, p. 440). The implications for the specific case at issue are clear but not its broader implications. Case law only provides a broad, general guideline and mostly refers to a general principle (Obermaier, 2009, p. 32) that Member States should apply on a case‐by‐case basis, considering the respective contextual factors. This gives leeway to Member States. How Member States can respond to the legal framework is explicated in the subsequent section.

Recently, since *Dano* (C‐333/13) and *Alimanovic* (C‐67/14), the ECJ has placed emphasis on residence conditions (O'Brien, 2017a, 2017b; Thym, 2015). It no longer mentioned factors such as unreasonable burden or a certain degree of integration but held that EU citizens had the right to equal treatment ‘*only* if their residence in the territory of the host Member State complies with the conditions of Directive 2004/38’ (*Dano*, para 69), which – in the case of economically inactive EU citizens – included that they had sufficient resources (Verschueren, 2015, pp. 370ff.).The Court has departed from the need to consider proportionality and to conduct an individual assessment. Nevertheless, Member States can still interpret EU law differently as will be shown in the following.

## II. The Effect of the ECJ on the Ground

### *Shades of Compliance*

**Figure 1:**
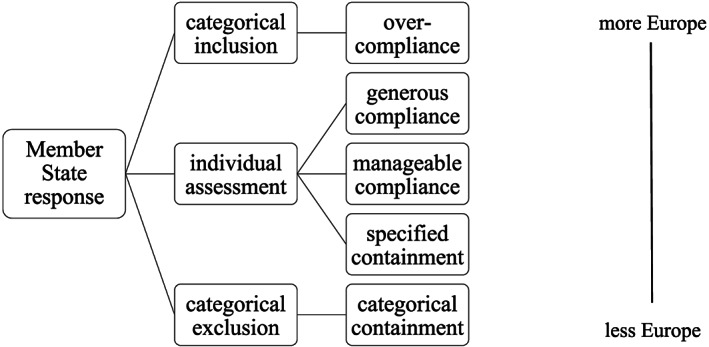
Member State Responses to ECJ Case Law on Social Citizenship.

Traditionally, scholars have differentiated between ‘compliance’ and ‘non‐compliance’ or ‘contained compliance’ with EU law (for instance Börzel, 2001; Conant, 2002; Falkner *et al.,* 2007). (Non‐)compliance is, however, rarely black and white and presenting it as a dichotomy masks an understanding of all the possible responses (Blauberger, 2012, p. 111; Thomann and Sager, 2017). While several researchers have overcome this dichotomy and described further degrees of ‘compliance’ (for instance Blauberger, 2012; Panke, 2007), this work provides a more nuanced framework, especially suited for ECJ case law on social citizenship that can be more distinctly perceived along the outcome‐dimension of ‘more versus less Europe’ and along the process‐dimension of categorical assessment versus individual assessment: Member States can, first, either make an individual assessment or a categorical assessment. Second, the individual or categorical assessment can be carried out in favour of more Europe or in favour of less Europe (see Figure 1).

Member States can thus react via instances of ‘(non‐)compliance’ which neglect the individual assessment per se and make either a categorical inclusion (more Europe) or exclusion (less Europe):

*Over‐compliance*: Member State administrations treat any request in an expansive, pro‐EU way and always grant rights, partly only demanding that EU citizens fulfil bureaucratic preconditions. This eventually leads to over‐compliance with ECJ case law as it entails concessions which the ECJ never demanded (Conant, 2002, p. 70). The Court has always left open the possibility for Member States to take the view that any specific request could be non‐proportional and that the respective Union citizen thus does not enjoy rights.
*Categorical containment*: At the other extreme, Member State administrations generally deny rights and thus severely minimize the impact of the ECJ by acting in favour of less Europe in a categorical way.

Between these two extremes, Member States can also make an individual assessment. Here, there are three options which vary on the outcome dimension:

*Generous compliance*: Member States take numerous but also vague factors as highlighted by ECJ case law (such as a degree of integration and unreasonable burden in terms of economically inactive EU citizens' welfare rights), into account, with a tendency to be favourable to the interest of the person concerned. The resultant individual assessment promotes more Europe with Member States presuming that EU citizens are eligible for rights. This is probably the preferred response envisioned by the ECJ.
*Specified containment*: At the other extreme when undertaking an individual assessment, Member States apply rigid, demanding criteria of eligibility which have no discernible link to ECJ rulings or interpret their criteria in a very restrictive way. Member States contain the impact of ECJ case law according to specified criteria and prevent that EU citizens are eligible for rights.
*Manageable compliance*: Between these two extremes, a third approach is to conduct the individual assessment according to few clearly defined, rigid criteria, under which individual rights are granted. In the case of welfare access for economically inactive EU citizens, this would for instance consist of applying one criterion which was decisive in one specific ECJ judgment and hence in one individual situation to any request they face. This manner of assessment benefits the authority insofar as handling cases becomes easier and more manageable than is the case when employing generous compliance. Member States try to harmonize their practice with ECJ case law and, at the same time, to bend it in order to promote or protect their own interests.

The responses are ideal types, meaning that Member State practices may still crossover from one response type to another. Nevertheless, the typology allows for a vast improvement in the analytical assessment of Member State reactions, describing their responses, changes in their responses, as well as for comparing between and partly within Member States.

### Social Citizenship at the Street Level: Responses of Member State Administrations

Conant (2002, p. 33) already identified ‘complete application as policy’ to be a rare outcome of Member States when reacting to ECJ case law. Moreover, scholars showed that the application of ECJ case law on social citizenship was increasingly guided by signals from domestic political and administrative superiors which reduced authorities' discretion and led to uniform restrictive practices across member states (Martinsen *et al.,* 2019). As I argue in this article, the impact of the ECJ is limited – also without signalling, and I emphasize that the responses are more faceted than Conant's overall concept of ‘contained compliance’.

Member States vary in terms of systems and structure, but in the end, their administrations share the same incentives. Member State responses thus need to be assessed in combination with the main goals and incentives of administrations in general and of street‐level bureaucrats in particular: as research on national implementation already held, the latter have the task to grant rights to certain persons and may try to meet the demands of their clients, but they nevertheless have to meet their internal demands (Tummers *et al.,* 2015): they have an incentive to keep, first, the administrative costs, and second, the financial costs low (Feldman, 2003, p. 281). First, street‐level bureaucrats face in general bureaucratic difficulties such as a lack of resources, namely workforce, time or information (Lipsky, 1969, pp. 4–12). They need to overcome the gap between demands and available resources and keep their work feasible. As a consequence, administrations need simplifying categories they can work with (Lipsky, 1969, pp. 4–12, 24; Hupe and Buffat, 2014, p. 551). Second, administrations have limited financial resources at their disposal. They are thus encouraged to economize expenditure (Brodkin, 1997, p. 24; Jewell, 2007). Granting individual rights is costly. As a consequence, there need to be criteria under which citizens are eligible for rights.

Administrations search for what they consider to be the best possible or most viable options that suit their interests and goals. *Generous compliance* is unlikely as it would incur a high administrative burden for Member States in terms of both workload and of legal ambiguity since case workers have to assess each request individually taking many factors which could justify ‘eligibility’ into account. Moreover, if rights are linked to financial costs, it would be costly in terms of finance since Union citizens would often be entitled to rights in the end. If interpreting ECJ case law in a generous way, there is another option which lightens the bureaucratic workload and which is thus more attractive: *over‐compliance*. This shade of compliance is easier to implement: case workers grant rights without individual assessments and essentially treat EU citizens as nationals. This response may, however, be questioned if high financial costs are involved and if this issue becomes salient.

Administrations may try to find solutions which entail fewer financial costs. *Manageable compliance* and *specified containment* bring with it a greater workload than the options of categorical containment and over‐compliance which do not involve any individual assessment at all. Having said that, they still generate a lesser workload than generous compliance as the eligibility criteria are clearly specified. The individual assessment process is made feasible and is only linked to financial costs if EU citizens meet the specific criteria. Specified containment implies the same workload as manageable compliance since it also involves an individual assessment according to clear criteria. It carries financial costs if EU citizens fulfil the respective preconditions, but given this would apply to lower numbers of persons than manageable compliance – due to the stricter criteria – the costs are similarly lower. *Categorical containment* finally implies low costs but may be challenged in court. In summary then, according to pure bureaucratic logic, one can reasonably expect to find any of the responses on the ground with the exception of generous compliance. 2This, however, cannot always be considered as the final word in the matter. One also has to take into account that decisions of street‐level bureaucrats can be (constantly) overturned by courts (Conant, 2002, p. 80). There may, of course, be exceptions. The explanations are, however, expected to capture the larger trend.

### Explaining the Varying Impact of the ECJ on the Ground

To which extent street‐level bureaucrats ‘comply’ with ECJ case law depends on different domestic factors which may vary between and also within Member States, and over time. As street‐level bureaucrats generally follow rules which are clear, easy and in line with the overall goal, it is decisive whether or not the vague provisions and terms of EU law are reflected in national laws or guidelines (Sampson Thierry and Martinsen, 2018; Martinsen *et al.,* 2019). If there are no *clear* rules, local authorities are *granted* discretion. How do authorities finally *use* this discretion?

Several scholars have claimed that the *party‐political environment*, namely the party constellation in the (local) legislature, has a significant impact on the decisions of street‐level bureaucrats. Hence, the leading (local) party is expected to have a high influence on ‘compliance’ as it may or may not exert adaptational pressure (for instance Keiser, 1999; Sack, 2012; Treib, 2004). Its orientation on a left versus right scale as well as on a *green/alternative/libertarian (GAL) versus traditionalism/authority/nationalism (TAN)* scale (Hooghe and Marks, 2008) is thus highly important: if the issue corresponds to the political aims of the predominant party, it pushes forward the implementation. In contrast, if the issue contradicts the political aims of the leading party in government, it may (exert pressure on administrations to) refuse to implement it or implement it incorrectly. The null hypothesis can thus be formulated as follows.

: The more EU friendly and the more expansive EU law fits the political aim of the predominant (local) party, the more likely ‘more Europe’ is on the ground. In contrast, the more EU‐sceptical the predominant (local) party, the more likely ‘less Europe’ is on the ground.

Other researchers highlighted the relevance of exposedness (Martinsen, 2005) which leads to the fact that authorities face a lot of (similar) requests and become therefore routinized. And they may see increasingly the need to tighten the rules and keep financial costs low (Brodkin, 1997, p. 24), namely to interpret ECJ case law restrictively. In contrast, if there is low (perceived) exposedness and thus less professional and routinized street‐level bureaucrats, it is likely that the case workers, managers or supervisors develop simplifying strategies for overcoming the special difficulty. This leads to Hypothesis 1.

: The more exposed the local authority, the more likely ‘less Europe’ is on the ground. In contrast, the less exposedness and therefore professionalization on the ground, the more likely ‘more Europe’ is on the ground.

Exposedness hence refers to whether the local entity has to deal with a low or high respectively strongly increasing number of requests of (EU) citizens. This increase or number is perceived by the case worker (as articulated in interviews) and also displayed in absolute numbers of (EU) citizens or mass bureaucracies. The two hypotheses will be tested in the subsequent empirical section.

## III. Empirical Analysis

I compare empirical evidence from Austrian authorities' practices: how – and why – did they apply ECJ case law on social citizenship? Austria is chosen as it allows for a within‐country comparison: Austrian laws mostly reflect the ambiguous and under‐specified character of EU rules, and administrations are thus granted discretion when applying ECJ case law. Yet there is variation within Austria concerning the explaining factors (see Table 1; details on the explaining factors will be presented in the case studies hereafter), which allows for testing the explaining factors via co‐variational analysis with regard to their explanatory power (Blatter and Haverland, 2014) The analysis is enriched through the methods of process tracing (Beach and Pedersen, 2013) which focuses on the causal chain between the explaining factor and the outcome of the dependent factor.

**Table 1:** Explaining factors

	**Austria: Vienna**	**Austria: other districts/regions**
**Predominant party's orientation**	*GAL, left*	*TAN, right* and *GAL, left* comparisons possible
**Exposedness**	*very high*	*low* and *high* comparisons possible

In order to examine how cross‐border welfare rights are handled in practice in Austria, I conducted 38 semi‐structured interviews mainly with case workers dealing with minimum benefits and those dealing with residence rights but also some additional interviews with rights advocacy groups, interest groups or within Ministries (see Table 2). The interviews with local authorities were decided upon carefully, based upon a representative and systematic sample. Those local authorities interviewed varied with regard to several factors: local and regional party‐political environment, urban versus rural areas, areas with a high relative respectively absolute number of EU citizens versus areas with a low relative respectively absolute number of EU citizens. Interviewees were asked how they handled the requests of EU citizens, why they did so, and, if applicable, why they changed the practices. Moreover, I analysed the respective laws, guidelines or manuals if applicable, as well as documents and websites of the authorities involved.

**Table 2:** Interviews conducted in Austria

	38 interviews
**Social & residence rights**	Social and immigration units of district administrations (Vienna: 2; other districts: 26)
	Social units of provincial governments (2)
**Additional interviews**	Rights advocacy groups (Vienna: 1; other districts: 1)
	Austrian‐wide interest groups/Ombudsman institution (2)
	Ministry of Social Affairs (2)
	Ministry of the Interior (2)

### Austrian Minimum Benefits and Residence Rules: Reflecting Vague EU Provisions

Austrian minimum benefits (former *Sozialhilfe*, nowadays *bedarfsorientierte Mindestsicherung, BMS*) are within the competence of the nine regions. With the introduction of BMS, they were partly harmonized at the federal level by a framework agreement (*BMS‐Vereinbarung*). All regional laws of former *Soziahilfe* and of BMS (with the exception of Vienna, see below) as well as the framework agreement reflect the vague character of EU law and do not provide specific rules about the access to social benefits; they link it to a right of residence but leave in the dark when this is exactly the case (*cf*. Windisch‐Graetz, 2014). Consequently, one also needs to determine the character of residence legislation.

According to the Residence and Settlement Act (*Niederlassungs‐ und Aufenthaltsgesetz*, NAG), economically inactive EU citizens are allowed to stay more than three months in Austria if they have sufficient resources and comprehensive health insurance so that they do not need to have recourse to neither social assistance benefits *nor the supplementary pension* during their period of residence (§ 51 (1) 2 NAG; 2009–2011; in italic: since 2011). Austria further took advantage of the possibility provided by EU law and introduced a registration certificate (*Anmeldebescheinigung*) which, under EU law, *documents* the right to reside, without constitutive character (Art. 8 Directive 2004/38). EU citizens who want to stay longer than three months have to apply for this certificate with the competent migration authority within the first four months of their stay. In order to be issued the certificate, economically inactive EU citizens have to prove their right of residence through evidence on their sufficient resources and health insurance (§ 53 (2) NAG). As regards the loss of right of residence, § 66 (1) Aliens Police Act (*Fremdenpolizeigesetz*) lays down that EU citizens can be expelled because they no longer meet the criteria necessary for right of residence or because they do not provide sufficient documentation for their right of residence. Austrian residence legislation is thus also characterized by under‐specified core concepts and ambiguity. It does not clearly define the criteria under which EU citizens enjoy a right of residence and under which they may lose it. In summary, both Austrian social and residence legislation leave room for interpretation to the administration.

Furthermore, there is no guiding Austrian‐wide institution for minimum benefits which provides instruments on how to assess requests. The social units of city and district administrations (*Magistrate* or *Bezirkshauptmannschaften*) which distribute minimum benefits are thus still given leeway. 3They are under the supervision of the social units of the regional governments (*Ämter der Landesregierungen,* respectively the *Magistrat* in the Viennese case) which do, however, mostly not provide guidelines. Vienna is a special case as it is both a city and a region.


### The Party‐Political Environment Cannot Account for Variation

According to the null hypothesis, namely that the party‐political environment matters, one would expect Vienna to have practices which imply more Europe: its, for decades, leading social‐democratic party SPÖ has a both left and *GAL* orientation. It is immigration‐, social assistance‐friendly and pro‐EU. For instance, in its programme for the regional elections in 2015, it declared itself in favour of ‘a social Europe and a Europe of solidarity’ and highlighted the importance of minimum benefits (SPÖ Wien, 2015, p. 11). In contrast, for instance in Tyrol, one would expect to find EU citizens’ access to social benefits restricted in a way which implied less Europe as its dominant centre‐right and moderate *TAN* party ÖVP was more sceptical towards immigration and social assistance.

These expectations are, however, not confirmed, as will be elaborated in detail in the subsequent section of this article. Viennese welfare authorities have started to categorically exclude economically inactive EU citizens from the entitlement to social assistance and thus *categorically contained* ECJ case law. Other larger cities partly also governed by social‐democratic parties followed. In other districts, irrespective of the regional or local party constellation, economically inactive EU citizens could have access to minimum benefits. There is thus no systematic relationship between the party‐political environment and the handling of EU citizens' access to social benefits on the ground. The null hypothesis can therefore be rejected. It is rather local exposedness that determines the shade of compliance as will be illustrated in the following.

### The Street Level: High Variation of Exposedness between Cities and Rural Areas

The number of (EU) claimants of minimum resources varies widely within Austria based on location: in contrast to welfare authorities in cities, in particular Vienna, those in rural areas do not face significant numbers of requests from (EU) citizens. To begin with, the regional numbers of all persons receiving minimum benefits, irrespective of the nationality, clearly illustrate some trends. First of all, Vienna has by far the most recipients of minimum benefits. In 2016, more than 173,400 persons received assistance; the other regions ranged between around 1,900 recipients (Burgenland) and around 30,600 (Lower Austria) (Pratscher, 2017, p. 12). These recipients corresponded to 0.65 per cent of the total population in Burgenland, to 1.84% of the total population in Lower Austria, and to 9.31 per cent of the total population in Vienna (Statistik Austria, 2017). Accordingly, Vienna also had the highest expenditure on minimum benefits, spending around €583 million in 2016, in stark contrast to the other regions whose expenditure ranged between €7 million in Burgenland to €73 million in Lower Austria (Pratscher, 2017, p. 20).

Considering regional figures, the assertion that benefit receipt is in general an urban phenomenon is confirmed. For instance in Tyrol, 7,470 persons received minimum benefits in 2016 in the city of Innsbruck (5.56% of all inhabitants), compared to 307 (Landeck; 0.69% of all inhabitants) and 4,381 (Innsbruck‐Land; 0.25% of all inhabitants) persons in the other districts. Around 45% of all recipients of minimum benefits in Tyrol in 2016 resided in the city of Innsbruck. Accordingly, the expenses for minimum benefits were also higher there (Amt der Tiroler Landesregierung, 2017, pp. 49, 53). From this data, one can conclude that cities are, first, in general engaged in mass procedures regarding minimum benefits and thus have greater incentive to keep administrative costs low, and, second, may have even more incentive not to increase the already relatively high financial costs.

With regard to EU citizens in particular, the picture is similar: in Vienna, around 13,000 EU citizens, out of a pool of 160,000 recipients in total, were granted minimum resources in 2014 (data received from *MA 40*). For example in Tyrol in January 2016, 326 EU citizens received benefits in the city of Innsbruck, compared to 0 in Landeck and 219 in Innsbruck‐Land (Tiroler Landtag, 2016). In total in Tyrol, 801 EU citizens drew benefits in 2016. This data further suggests that case workers in cities are more familiar with requests of EU citizens, and that they may see the need to tighten vague and ambiguous rules for EU citizens more than rural areas, keeping the financial costs in mind.

### Less Exposed Welfare Authorities

As interviewees in rural areas indicated, they still perceived EU citizens to be a low number of claimants (interviews with welfare authorities, October 2015), which is exactly what the data confirmed. As a consequence, case workers in welfare authorities had little experience and were certainly not specialists in minimum benefits for EU citizens, and especially not in residence rights and EU law. Most interviewees mentioned that their primary instrument to assess the requests was the relevant regional law; EU law did not play a big role (interviews with welfare authorities, October 2015). The respective provisions of the regional laws were, as already demonstrated, vague and complex. In the beginning of the period that was analysed, some local authorities thus treated benefit requests of EU citizens as those of Austrians and hence generally granted them access to minimum resources (interview with welfare authority, October 2016). By not making an individual assessment at all but simply opening the door to the benefit, those authorities were more generous than the ECJ ever demanded and thus *over‐complied* with the Court's case law.

These generous practices of some authorities were, however, abandoned over time. As exposedness increased with growing numbers of EU citizen application, as interviewees explained, authorities began searching more earnestly for rules and ways to curb access. Yet, supervisors in the superior authority were aware of the complexity of the law, and gave simplifying advice in order not to overburden case workers. Supervisors insisted on a separation of competences between social authorities and migration authorities because the latter were competent in residence rights (interview with social unit of provincial government, January 2016). They told the welfare case workers to leave the assessment of lawful residence, the precondition for the access to social benefits, to the migration authority and thus to rely chiefly upon the registration certificate. The registration certificate was, as several case workers pointed out, the ‘linchpin’ around which everything now revolved (interviews with welfare authorities, October 2015): welfare authorities granted the benefit as soon or as long as EU citizens had the certificate and basically treated the certificate as constitutive. They did not make a case‐by‐case assessment but generally granted minimum resources if the EU citizen fulfilled the bureaucratic precondition and had the right form. The opposite was also true; they generally denied benefits if EU citizens could not prove their right of residence via the registration certificate (interviews with welfare authorities, October 2015).

This practice went simultaneously beyond what the ECJ ever demanded and against its intent. On the one hand, case workers *over‐complied* with EU law when not questioning lawful residence because of over‐valuing the certificate of the EU citizen at the point of application for benefits and simply relying on the form. On the other hand, they *categorically contained* the impact of ECJ case law when neglecting the independent status of EU citizens, namely such citizens that met the criteria for lawful residence but did not have a registration certificate were denied the access to benefits. In the case of over‐compliance, one needs to take a closer look at the assessment of migration authorities when issuing the certificate to (newly arrived) EU citizens in order to finally determine the shade of compliance of Austrian practices with regard to EU citizens' access to minimum benefits.

While residence legislation is vague as well, residence policy is characterized by a more centralized structure than minimum resources as residence policy is set by the Federal Ministry of the Interior. The latter provides a manual for the various local migration authorities of the local offices on how to deal with the Residence and Settlement Act and offers regular training courses. According to the manual, ‘sufficient resources’ meant that people did not claim social assistance benefits (Bundesministerium für Inneres, 2006, p. 187). After the legal reform of the Residence and Settlement Act with regard to the supplementary pension, the manual suggested *orienting the baseline* to the standard rates of minimum benefits or the supplementary pension (Bundesministerium für Inneres, 2015, pp. 11f.).

In practice, migration authorities gladly seized upon these indicators and used the standard rate of minimum benefits, and later also supplementary pension, as a reference point (interviews with migration authorities, October, November 2015, January 2016). Having received some clarity regarding one aspect of this issue, case workers were still confronted by uncertainty over another. Due to a lack of clear rules regarding the timeframe for which EU citizens needed to have sufficient resources as well as the nature of the resources, authorities established their own criteria. In the beginning of the period that was analysed, economically inactive EU citizens who could prove that they had means equivalent to the standard rate of minimum benefits for some time were able to receive the registration certificate which finally allowed them to draw benefits. For instance, EU citizens could prove they had ‘sufficient resources’ by means of bank documents showing a reasonable amount of savings (interviews with migration authorities, October, November 2015, January 2016). In short, since the manual did not lay down precise rules, migration authorities made a case‐by‐case assessment, but had their own thresholds of ‘sufficient resources’ regarding the period of time for which they had to be available. They followed EU law by and large when orienting themselves on the standard rate of minimum benefits; however, in developing criteria regarding the period of time, migration authorities selected a period that made the assessment feasible. Austrian migration authorities reacted via *manageable compliance*. As there were no clear (legal) rules, the criteria differed from district to district. Consequently, local practices varied in their shades of manageable compliance.

Over time, triggered by rising numbers of EU citizens, perceptions of ‘abuse’ coupled with feedback from the welfare authorities, namely the fact that EU citizens had access to social benefits once they had received the certificate, migration authorities finally gained more experience and somewhat daringly decided that they needed to interpret the criteria more restrictively (interviews with migration authorities, October, November 2015): the first step in this regard was that economically inactive Union citizens had to prove they possessed sufficient resources for a longer period of time in order to receive the certificate. EU citizens needed so much money that they could live from it for several years: interviewees mentioned that these persons had to have available liquid assets amounting to the equivalent of the standard rate payments for a period of one to three years (interviews with migration authorities, October, November 2015, thus on average for two years. In 2016, this amounted to around €20,000 (standard rate of BMS) or €21,000 (standard rate of supplementary pension). The second step taken in reinterpreting and tightening criteria saw migration authorities no longer accept various kinds of resources: in most districts interviewed, saving deposits were only accepted if EU citizens could prove that it was their money and not just transferred into their bank account for the purpose of the registration certificate. As a consequence of this overtly high threshold, EU citizens had to be relatively rich, or needed to be either a worker or self‐employed in order to receive the certificate. In summary then, migration authorities still made an individual assessment, but according to strict criteria in terms of less Europe which could not reasonably be met by economically inactive EU citizens, thus limiting the influence of EU law via *specified containment*. Despite its declaratory character, the registration certificate ultimately served to exclude most EU citizens other than workers and self‐employed persons from any entitlement to benefits. Most welfare authorities used to grant minimum resources to all EU citizens showing their registration certificate, but this over‐compliance by the welfare authority was counteracted at first by manageable compliance and then, with rising exposedness, by specified containment at the migration authority.

Nevertheless, the procedure of separate competences still at times led to consequences which were more generous than intended (Heindlmaier and Blauberger, 2017). EU citizens could fulfil the conditions while economically active and be issued the certificate, but then draw minimum benefits afterwards when no longer working. Such persons were “only” in danger of losing their right of residence. The registration certificate once issued was very rarely revoked (for data on expulsion measures see Nationalrat, 2014). This led highly exposed welfare authorities to change the practices and leave the procedure of separate competences aside as will be presented hereafter.

### Highly Exposed Authorities

Welfare authorities in cities that faced a high number of requests in general or of EU citizens in particular, notably Vienna, had ever increasing incentive to keep the costs low, and thus examined the eligibility themselves and independently tightened the rules. First and foremost local managers engaged with the law and questioned the practices in force as well as the current interpretation of legal rules and established criteria for the eligibility assessment. This assessment of the criteria themselves, triggered by high exposedness and a desire to economize on welfare expenditure in mind, resulted in them eventually becoming stricter (interviews with welfare authority, January 2016, with interest group January 2016, with the Ministry for Social Affairs, January 2016). As interviewees stated, they interpreted the fact that EU citizens sought to claim benefits as a proof that they no longer fulfilled the precondition of sufficient resources and were thus not lawfully residing (interviews with rights advocacy group, November 2015, with welfare authorities, January, October 2016). They *categorically contained* the impact of ECJ case law.

The step towards assessing lawful residence was of course a challenge for case workers and initially confusing. Therefore, case workers received training and some city authorities also created internal guidelines that workers could build upon when evaluating the situation of EU citizens (interviews with welfare authorities, January, October 2016). In the Viennese case, this exclusion happened as early as 2007 (Magistratsabteilung 15, 2007, pp. 53 f.). The other large urban centres in Austria began to follow this same restrictive approach later when having learnt that their procedures were not able to adequately address the benefit requests of EU citizens (interview with welfare authority, October 2016). Highly exposed welfare authorities thus categorically contained compliance even in their instruments, helping to keep both administrative as well as financial costs low. In Vienna, the step towards a categorical exclusion was eventually also taken at the legislative level in 2010 (§ 5 (2) *Wiener Mindestsicherungsgesetz*).

To sum up, Austrian welfare authorities' practices regarding the assessment of benefit requests of EU citizens differed largely: whereas most authorities built upon the registration certificate and granted minimum benefits as soon or as long as EU citizens had this form, some authorities have started to assess the precondition for benefit receipt, namely lawful residence, themselves. These two varying strategies can be attributed to differences in exposedness. The scenario of separate competences kept the administrative burden for welfare authorities low as they relied heavily on the registration certificate which served to exclude most economically inactive EU citizens from the social assistance system. Still, this procedure was only likely to persist if financial costs remained relatively low, meaning that few people drew benefits. In this case, financial costs did not outweigh administrative costs; over‐compliance was thus not deemed as too costly, a situation that stands in stark contrast to highly exposed authorities. If exposed, authorities gained knowledge, had to recalculate the administrative and financial costs, and thus felt the need to respond more restrictively to ECJ case law.

## Conclusion

By studying the application of ECJ case law on social citizenship by Member States' local authorities (which are granted discretion), this contribution has addressed three gaps in research: First, in order to describe and compare Member State practices, a typology of shades of compliance was developed, which goes beyond the prevailing dichotomy of compliance and non‐compliance. This typology allows for a more nuanced assessment of Member State practices in the light of ECJ case law and demonstrates to what extent the impact of the ECJ is limited – while the ECJ required Member States to assess the benefit claims of economically inactive EU citizens on a case‐by‐case basis by taking factors such as a degree of integration into account (what I call *generous compliance*), Austrian administrative practices have differed and limited the Court's influence (this finding is confirmed for other Member States by Kramer *et al.,* 2018). Austrian authorities have interpreted EU law in a way which implies less Europe (from *manageable compliance* to *specified containment* and partly to *categorical containment*) and have set a firewall. Consequently, at the street level, there is no ‘social citizenship’ as it was probably envisioned by (expansive) ECJ case law – and with recent case law of the Court reacting to the broader political mood (Blauberger *et al.,* 2018) from *Dano* on, it has only become easier for Member States to handle EU citizens' social rights in a way of less Europe.

Second, my contribution adds to existing research as it provides an argument for why authorities tend to limit the impact of the Court if it is linked to costs, independent from signals from domestic superiors (Martinsen *et al.,* 2019): Limited social citizenship can be explained by the fact that, in the end, administrations share the same rational incentives, to keep both administrative and financial costs low. While there is a broader trend to limit the influence of EU law in practice, there are differences in the shade of compliance on the ground. As this contribution shows, third, this varying impact of the Court at the street level cannot be explained by the respective party‐political environment, 4While there is room for politics in the ECJ case law implementation process (Martinsen *et al.,* 2019), this signaling, in form of laws for instance, also cannot be explained by the party‐political environment. but depends upon the degree of exposedness: This research has identified numerous restrictive practices that occur independently from the party preferences of given political context (at the national, regional and local level).

In terms of policy relevance, this article confirms that ECJ case law is a burden for national administrations in terms of workload, as Blauberger and Schmidt already highlighted. According to earlier ECJ case law, administrations would need to apply broad principles and carry out case‐by‐case assessments (Blauberger and Schmidt, 2017). Conducting individual assessments, at least without fixed criteria, is hardly feasible for administrations that have to deal with a flood of procedures given their limited administrative resources. While ECJ case law was to some degree categorically contained, Member States at times undertook individual assessments but specified the criteria of eligibility in order to make this task feasible. From a policy perspective, the article thus illustrates case workers' demand for clear rules. While recent case law no longer pointing to proportionality has lightened the burden for authorities, it has confirmed the exclusion of economically inactive EU citizens from welfare rights. In summary then, the social rights of economically inactive seem to be quite settled, in a way which implies less Europe.

## Appendix

**Table:**

Interview number	Organization	Position	Date
AT 1	Social unit of district administration 1	Head of division	7 October 2015
AT 2	Migration unit of district administration 1	Head of division	7 October 2015
AT 3	Social unit of district administration 2	Head of division	7 October 2015
AT 4	Migration unit of district administration 2	Case worker, consultant	8 October 2015
AT 5	Social unit of district administration 3	Case worker, consultant	12 October 2015
AT 6	Social unit of district administration 4	Case worker, consultant	13 October 2015
AT 7	Social unit of district administration 5	Head of division	13 October 2015
AT 8	Social unit of district administration 6	Case worker, consultant	20 October 2015
AT 9	Social unit of district administration 7	Head of division	20 October 2015
AT 10	Social unit of district administration 8	Case worker, consultant	21 October 2015
AT 11	Social unit of district administration 9	Head of division	22 October 2015
AT 12	Social unit of district administration 10	Head of division	23 October 2015
AT 13	Social unit of district administration 11	Head of division	27 October 2015
AT 14	Migration unit of district administration 3	Case worker, consultant	28 October 2015
AT 15	Social unit of district administration 12	Head of division	28 October 2015
AT 16	Migration unit of district administration 4	Case worker, consultant	29 October 2015
AT 17	Social unit of district administration 13	Case worker, consultant	29 October 2015
AT 18	Social unit of district administration 14	Head of division	30 October 2015
AT 19	Migration unit of district administration 5	Case worker, consultant	2 November 2015
AT 20	Migration unit of district administration 6	Case worker, consultant	2 November 2015
AT 21	Social unit of district administration 15	Head of division	3 November 2015
AT 22	Migration unit of district administration 7	Head of division	11 November 2015
AT 23	Migration unit of district administration 8	Case worker, consultant	17 November 2015
AT 24	Rights advocacy group	Social worker + advisor	19 November 2015
AT 25	Social unit of district administration 16	Policy officer + head of division	19 November 2015
AT 26	Social unit of provincial government 1	Policy officer	16 December 2015
AT 27	Social unit of provincial government 2	Policy officer	12 January 2016
AT 28	Migration unit of district administration 9	Consultant	25 January 2016
AT 29	Social unit of district administration 17	Head of division	26 January 2016
AT 31	Ministry of Labor, Social Affairs and Consumer Protection	Head of department	27 January 2016
AT 32	Interest group/rights advocacy group	Policy officer	27 January 2016
AT 33	Ministry of the Interior	Policy officer	28 January 2016
AT 34	Ombudsman institution/rights advocacy group	2 policy officers	28 January 2016
AT 35	Ministry of the Interior, Federal Office for Immigration and Asylum	2 policy officers	1 September 2016
AT 36	Ministry of Labor, Social Affairs and Consumer Protection	Head of division	1 September 2016
AT 37	Rights advocacy group	Advisor	2 September 2016
AT 38	Social unit of district administration 18	Head of division	3 October 2016
